# Improved characterization and translation of NK cells for canine immunotherapy

**DOI:** 10.3389/fvets.2024.1336158

**Published:** 2024-02-06

**Authors:** Aryana M. Razmara, Alicia A. Gingrich, Christine M. Toedebusch, Robert B. Rebhun, William J. Murphy, Michael S. Kent, Robert J. Canter

**Affiliations:** ^1^Department of Surgery, University of California Davis School of Medicine, Sacramento, CA, United States; ^2^MD Anderson Cancer Center, University of Texas, Houston, TX, United States; ^3^Center for Companion Animal Health, Department of Surgical and Radiological Sciences, School of Veterinary Medicine, University of California, Davis, Davis, CA, United States; ^4^Department of Dermatology, University of California Davis School of Medicine, Sacramento, CA, United States

**Keywords:** NK cell, canine (dog), immunotherapy, clinical trials, markers

## Abstract

The field of cancer immunology has seen a meteoric rise in interest and application due to the discovery of immunotherapies that target immune cells, often leading to dramatic anti-tumor effects. However, successful cellular immunotherapy for solid tumors remains a challenge, and the application of immunotherapy to dogs with naturally occurring cancers has emerged as a high yield large animal model to bridge the bench-to-bedside challenges of immunotherapies, including those based on natural killer (NK) cells. Here, we review recent developments in the characterization and understanding of canine NK cells, a critical springboard for future translational NK immunotherapy research. The characterization of canine NK cells is exceptionally pertinent given the ongoing challenges in defining them and contextualizing their similarities and differences compared to human and murine NK cells compounded by the limited availability of validated canine specific reagents. Additionally, we summarize the current landscape of the clinical and translational literature employing strategies to capitalize on endogenous and exogenous NK cell immunotherapy in canine cancer patients. The insights regarding efficacy and immune correlates from these trials provide a solid foundation to design and test novel combinational therapies to enhance NK cell activity with the added benefit of motivating comparative work to translate these findings to human cancers with extensive similarities to their canine counterparts. The compilation of knowledge from basic canine NK phenotype and function to applications in first-in-dog clinical trials will support the canine cancer model and enhance translational work to improve cancer outcomes for both dogs and humans.

## Introduction

The advent of immunotherapy has propelled the field of oncology beyond the standard therapies of surgery, radiation, and chemotherapy. While the vast majority of successful immunotherapy methods to date have been T-cell based, such as PD-1/PD-L1 inhibition and CAR-T cells, such strategies are not universally successful in all patients. Thus, researchers have broadened their focus to harness and manipulate other immune cell types. Of these, natural killer (NK) cells have emerged as an attractive candidate given their innate cytotoxicity against a diverse array of targets and their potent cytokine production. NK cells are considered sentinels of the innate immune system, capable of identifying and killing virus-infected and cancer-transformed cells via mechanisms that do not require antigen-specific recognition.

NK cells have been a focus of potential therapy for decades, since initial trials by Rosenberg et al. in the 1980s, among others (1–3). However, severe toxicity was seen in early attempts, largely due to amplified cytotoxic lymphocyte responses and the concomitant use of high-dose IL-2 to support adoptive transfer of these cells into patients. These findings emphasized the need to minimize such responses while still harnessing NK cells’ anti-tumor effects. Companion canines have emerged as a useful model for studying novel cancer therapies given that dogs are a large, outbred species which develop spontaneous cancers in the setting of an intact immune system. To study NK cells in particular, the canine model is invaluable since the complex interplay between neoplasia development and a functional immune system can be evaluated. Here, we review recent canine immunotherapy trials which directly or indirectly act via NK cells while also summarizing the progress made and hurdles which still exist to advance canine NK immunotherapies.

## Identification and characterization of canine NK cells

Populations of innate lymphoid cells (ILCs) have been extensively studied in humans and mice for decades. By comparison, canine ILCs are less defined, although recent studies have sought to advance our understanding (4). The identification and clarification of the canine NK cell populations based on surface markers has been a longstanding effort (5–8). Early work established that they are CD4-/CD20-, as these are the canine T and B cell markers, respectively (9). A detailed review regarding the evolution of the collective understanding of canine NK cell identification was published by Gingrich et al. (10).

To summarize, many early efforts focused on phenotypic identification of canine NK cells using surface markers, as such markers differ from those in humans and mice (10). Huang et al. were the first to describe canine NK cells using the surface density of the CD5 marker, a member of the scavenger receptor cysteine-rich superfamily and typically classified as a T cell marker (11). This study noted important differences in lymphoid phenotype based on CD5 density, as cells with CD5^dim^ expression were larger, contained more cytoplasmic granules, and demonstrated antigen-independent cytotoxicity, especially in the setting of IL-2 enrichment (11).

Further studies by Shin et al. continued to focus on the density of the CD5 receptor as an NK marker, particularly contrasting CD3+CD5^dim^CD21− with CD3+CD5−CD21− cells (12). Following expansion and co-culture with K562 feeder cells and cytokines for 21 days, CD5^dim^ expressing cells did not express TCRαβ nor TCRγδ (12). Additionally, CD3+CD5^dim^CD21− cells exhibited significantly higher IFN-γ cytokine production compared to CD3+CD5−CD21− (12). Based on these findings, the authors proposed that each population represents canine NK cells at different levels of maturation (12), although the stages of canine NK cell maturation and development remain a poorly understood topic in contrast to key discoveries in mouse and human studies (13–15).

The “pan-mammalian” NK cell receptor, NCR1/NKp46, has also been identified as a marker of canine NK cells (10). Studies by Grondahl-Rosado et al. noted that CD3-NCR1+ cells comprised 2.5% of canine PBMCs, a proportion much lower than NK cells seen in other mammals (6, 7). Foltz et al. developed a novel antibody to canine NKp46 for use in flow cytometry (5). Their work also identified a CD3-NKp46+ NK subset, representing approximately 2–3% of PBMCs (5). These cells were found to be highly cytotoxic against multiple canine cancer lines. Using a novel expansion technique, the authors also identified a population of CD3+TCR+NKp46+ cells (5). The CD3 positivity in canine NK cells was postulated to represent a different stage of maturation, although a conclusive trajectory has not been described to date (5, 10).

More recently, Grudzien et al. established a canine NK cell line (CNK-89) derived from a dog with NK cell neoplasia (16). This cell line is CD5+CD8+CD45+CD56+CD79a+NKp46+. Although CD79a is classically a B cell marker, the presence of the NKp46 protein and mRNA expression of NKG2D, NKp30, NKp44, NKp46 and perforin suggested NK cell properties for this cell line. Following treatment with IL-12, IL-15, IL-18 and IL-21, increased expression of granzyme B, perforin and CD16 was observed (16). Secretion of TNFα and IFNγ was also noted. These findings were not observed following treatment with IL-2, suggesting these neoplastic-derived cells are an IL-2 independent cell line and potentially useful for studying alternate pathways of canine NK cell activation (16).

Gingrich et al. detailed differential gene expression analyses of the two most widely accepted canine NK cell populations: CD3-CD5^dim^ and CD3-NKp46+ cells (17). Marked differences were seen in steady-state cells, including non-detectable mRNA expression of granzyme B, perforin, IFNγ and KLRD1/CD94 in CD3-CD5^dim^ cells, but detectable expression in CD3+NKp46+ cells (17). Remarkably, following co-culture with radiated human feeder cells [K562cl9, (18)], the two cell populations converged on a nearly identical mRNA expression phenotype (17). The findings suggested each population likely contains NK cells that are selected for rapid and dominant growth under stimulatory co-culture conditions.

The authors then conducted single-cell RNA sequencing of FACS-sorted CD3-CD5^dim^ and CD3-NKp46+ cells to explore overlap between the two populations. In these studies, at steady-state the CD3-CD5^dim^ population was found to be more heterogeneous than the CD3-NKp46+ one. Gene expression driving the variance for CD5^dim^ cells was predominantly non-NKC gene expression, reinforcing that CD5^dim^ appears to be a less specific marker. Further single-cell studies following the activation of the two cell populations in co-culture demonstrated a conserved trajectory to activation based on uniform, discreet changes in gene expression in canonical NK transcription factors as well as marked changes in expression of granzyme A, IL2RB, and KLRB1. These data described the transition in both CD3-CD5^dim^ and CD3-NKp46- canine NK cells from a resting state to an activated state which may lend insight to the stages of NK cell maturation in dogs, a physiologic process which has yet to be clearly elucidated.

Overall, the precise identification of canine NK cell populations remains elusive, likely in part due to a lack of understanding of the maturation process as well as variable gene expression and protein surface markers associated with different stages of development. However, based on the studies above, populations of innate, canine lymphocytes capable of cytokine-dependent, antigen-independent cytotoxicity have been demonstrated to exist, paving the way for clinical applications of NK-based immunotherapies in dogs.

## NK cells in canine immunotherapy

**Figure 1:**
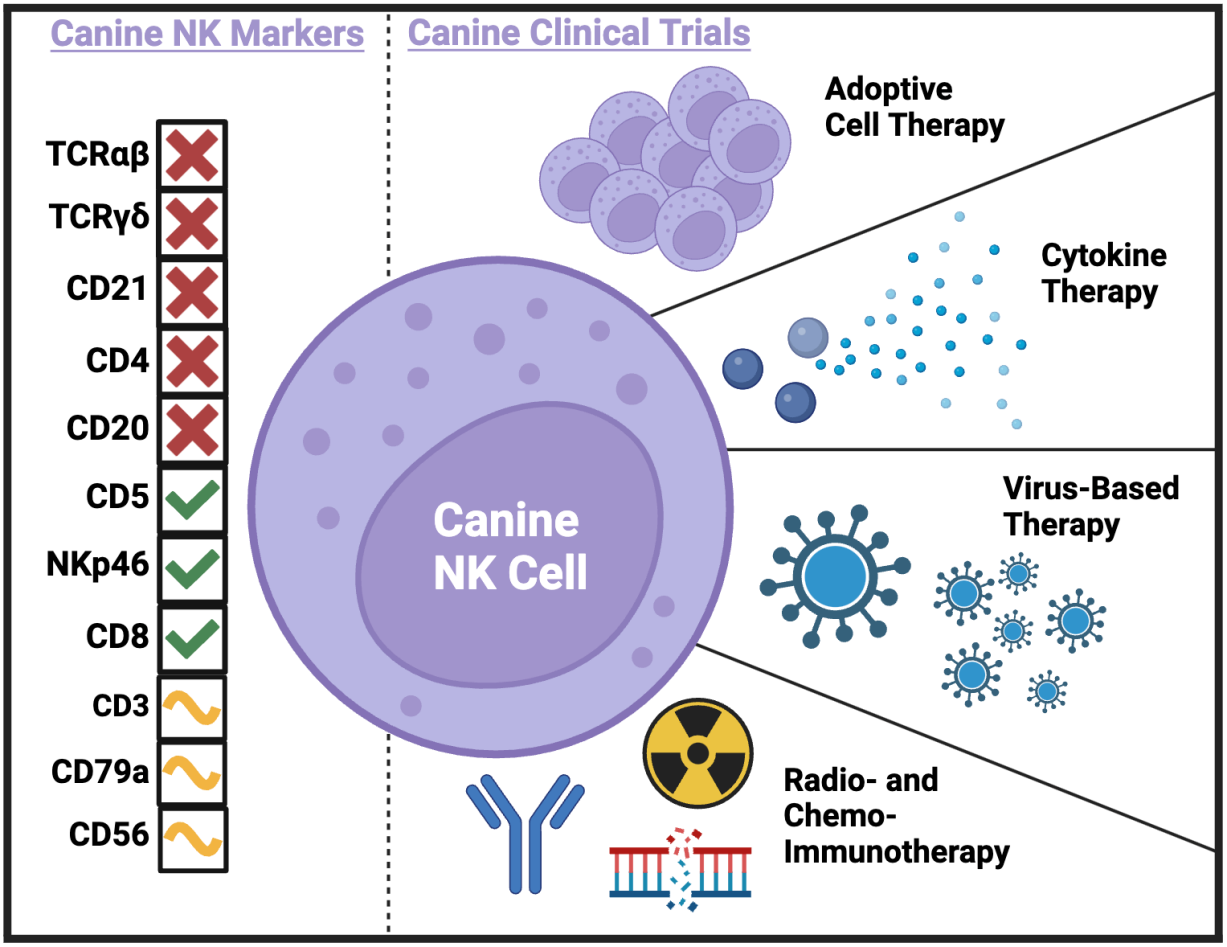
Current and potential canine NK markers and canine clinical trials completed to-date with NK cell correlates. Created with BioRender.com.

Ultimately, immunotherapy needs to be tested in immunocompetent hosts. This underscores a strength of the dog model, especially when novel immunotherapies are combined with serial immune correlates (19). The investigation of immune populations before, during, and following immunotherapy can not only provide insight to the presence or absence of clinical benefit in the relevant study but can also be hypothesis generating in the pursuit of improving efficacy. Additionally, immune correlates can bring to light potential biomarkers of response, leading to improved selection of dogs that are likely to respond to treatment and the identification of canine patient subsets that require innovative interventions. A concerted effort to combine the lessons learned from canine clinical trials performed or in progress is essential to the future of the field. To date, several canine immunotherapy trials have been completed with either direct or indirect effects on putative NK cell populations. Trials have used adoptive cell therapy, cytokine therapy, virus-based therapy, radio- and chemo-immunotherapy, and checkpoint blockade to treat dogs with spontaneous cancers with various methods of NK cell identification and analysis (Figure 1 and Table 1).

**Table 1:** Canine clinical trials with NK immune correlates.

General therapy	Specific therapy	Cancer diagnosis	NK correlates	Method of analysis	References
Adoptive cell therapy	Autologous NK cell transfer	Osteosarcoma	Number of NK cells	Flow cytometry	(20, 30)
			Serum cytokines	ELISA	
			Activation markers	Flow cytometry	
			Gene expression	qPCR	
Cytokine therapy	Inhaled IL-15	Osteosarcoma, melanoma	Number of NK cells	Flow cytometry	(31, 17, 22)
			Serum cytokines	ELISA	
			Activation markers	Flow cytometry	
			Gene expression	RNA sequencing	
			Cytotoxicity	Killing assay/flow cytometry	
Virus-based therapy	Oncolytic virus	Carcinoma, Adenocarcinoma	Number of NK cells	Flow cytometry	(24)
	Genetically modified HSV	Glioma	Gene expression	RNA seqencing/nanostring	(25)
	eCPMV	Inflammatory mammary cancer	Gene expression	RNA seqencing/nanostring	(27)
	eCPMV	Mammary cancer	Gene expression	RNA sequencing	(33)
			Number of NK cells	Flow cytometry	
	Cellular virotherapy	Various	Number of NK cells	Flow cytometry	(34, 35)
			Gene expression	RNA seqencing/CIBERSORT	
Radio- and chemo-immunotherapy	RT, TRT, IT-IC	Melanoma	Number of NK cells	Flow cytometry	(37)
			Gene expression	RNA sequencing	
	Chemotherapy, anti-CD20, SMI	B cell lymphoma	Gene expression	RNA sequencing	(38)

HSV, Herpes Simplex Virus; eCPMV, Empty Cowpea Mosaic Virus; RT, Radiotherapy; TRT, Targeted Radionuclide Therapy; IT-IC, intratumoral immunocytokine; SMI, Small Molecule Inhibitor.

Our group has completed several first-in-dog trials using adoptive NK cell transfer to treat dogs with spontaneous cancers. In 2017, we treated dogs with unresectable limb osteosarcoma (OSA) using palliative radiotherapy (RT) and two intratumor injections of autologous NK cells (20). NK cells were expanded from CD5-depleted PBMCs over a 14 day co-culture with irradiated K562-C9-mIL21 feeder cells and 100 IU/mL recombinant human IL-2 (21). We observed a significant increase in CD45+GZMB+ cells in PBMCs post-treatment by flow cytometry suggesting systemic immune effects of the treatment, although there did not appear to be an association between survival and frequency of GZMB+ or IFNγ+ cells in peripheral blood (30). Given the intra-tumor route of administration, we also analyzed tumor biopsies by flow cytometry and observed that approximately 50% of intratumor CD45+ cells stained positive for an intracellular dye label consistent with persistence of the adoptively transferred NK cells for one-week post-transfer in the tumor microenvironment (TME) (20). We analyzed tumor tissue by qPCR and showed gene expression varied greatly by patient, with no difference in fold change gene expression between dogs that were alive or dead at 6 months (30). Though, it is interesting to note that the longest surviving dog, 18 months, showed the greatest fold-change in the expression of CD3, CD8, and IDO1 genes following RT and intratumor NK transfer (30).

Immunotherapies can also stimulate endogenous NK cells through cytokines that are responsible for the activation, migration, and expansion of NK cells *in vivo*. Our group also completed a first-in-dog dose escalation trial in dogs with pulmonary metastatic melanoma and osteosarcoma using inhaled recombinant human IL-15 to stimulate NK cells in the lung at metastasis sites (31). Seven of the initial enrollees were also analyzed in a preliminary assessment of peripheral NK cells using flow cytometry and RNA sequencing (17, 22). The proportion of total NK cells and NK cells expressing Ki67 increased during inhaled IL-15 treatment and had a significant increase in Granzyme B fold change (22). Conversely, there was evidence of upregulation of TIGIT gene expression, an inhibitory marker, at both day 7 and 14 post enrollment (22). The increase in both Granzyme B and TIGIT suggests concurrent stimulation of activating and inhibitory pathways, the balance of which potentially determines response to treatment. RNA sequencing of patient PBMCs offers preliminary evidence that the activating/inhibitory balance may be patient specific, since principal component analysis (PCA) variance was driven largely by two dogs that responded to treatment (17). At the completion of trial, among 21 dogs total, we observed a 39% clinical benefit rate (31). Cytotoxicity of patient PBMCs against osteosarcoma (OSA) and melanoma (M5) targets significantly increased from pre- to post-therapy and maximal cytotoxicity was significantly correlated with patient survival (31). The finding of increased peripheral blood cytotoxicity across the entire cohort post-treatment suggests that tumor cell death is occurring, but only leading to improved survival in certain patients.

There are many immunotherapies that are not traditionally NK-targeting or are non-specific in nature, which still result in NK activation, making them attractive candidates for multimodal treatments (23, 32). For instance, oncolytic viruses are a unique type of immunotherapy in that their primary function is to invade and replicate within cancer cells, leading to lysis, but it was soon recognized that this process also increases the immunogenicity of cancer cells, leading to the recruitment of and elimination by immune cells. This is a similar mechanism through which viruses, like cowpea mosaic virus, are used as therapy to bind non-specific receptors and stimulate the induction of an immune response in the TME.

Martín-Carrasco et al. tested an intratumor oncolytic virus based on canine wild-type adenovirus which was engineered to selectively replicate in mutated cells to treat canine patients with cancer (24). The strength of the study was the availability of serial sample collections from patients before and up to one year after treatment. At least four of the eight patients had an increase in peripheral NK cells within the first month after treatment as assessed by flow cytometry (24). However, CD56 was used as the identifying marker, which is not known to be expressed on canine NK cells, highlighting the importance of validating both the reagents used as well as the underlying biology given the extensive cross-species differences in NK cells.

In another trial, the authors treated canine oligodendroglioma and astrocytoma using intratumor injections of M032, a genetically modified herpes simplex virus. The authors observed enrichment of tumor mRNA gene signatures associated with NK cells in four of six patients with available specimens (25). In this study, NK cell gene signatures were labeled as belonging to “NK CD56dim cells” and assessed by the NanoString Technologies gene expression panel (25). The classification of NK cells as CD56dim by this method is described as based on expression of IL21R in an evaluation of nearly 10,000 samples from The Cancer Genome Atlas (TCGA) (26). So, while CD56 or CD56^dim^ are not validated as canine NK markers, IL21R is thought to be expressed on canine NK cells and capable of being activated by its respective IL-21 ligand.

The same immune profiling method was used to investigate the abundance of NK cells in the TME of dogs with canine inflammatory mammary cancer treated with intratumor delivery of immunotherapy using empty cowpea mosaic virus-like particles (eCPMV) (27), which are recognized by toll-like receptors (TLRs). They found no significant change in cells labeled as “NK CD56dim cells,” or cells enriched for IL21R, and the fold change of other genes associated with NK cells including KLRA1, KLRD1, and GZMB were increased in treated tumor tissue, but not significantly (27). Conversely, there was significant upregulation in treated versus untreated tumor tissue of IL18R1 and significant downregulation of IL12RB2, which are both implicated in NK cell functions (27), supporting the pattern across canine immunotherapies in which both activation and inhibition are observed simultaneously. Another study treated patients with canine mammary cancer using eCPMV (33). This trial gave two injections into the largest tumor followed by tumor resection. In line with findings from dogs treated with eCPMV alone, NK-related genes were not differentially expressed using RNA-seq to analyze the tumors treated with neoadjuvant immunotherapy. Additionally, flow cytometry was used to define CD45+CD21−CD3−GZMB+ PBMCs as NK cells. This analysis showed insignificant changes in peripheral NK cells in response to eCPMV and surgery (33).

To bypass hurdles associated with non-surgical tumors and improve systemic effects, autologous canine mesenchymal stem cells can be infected with a canine oncolytic adenovirus and administered to patients. This was performed on 27 dogs with extracranial cancer and assessed by the same group in 10 subsequent dogs with high-grade gliomas (34, 35). In the initial trial, peripheral immune cells including NK cells increased after each dose, although the changes were not statistically significant (34). In the subsequent trial, dogs with gliomas received eight weekly treatments of cellular virotherapy (35). Using CIBERSORT analysis of bulk-RNAseq on tumor tissue, they found that NK cell fractions were not changed between responders and non-responders post-treatment (35).

Together, these virus-based therapies have shown preliminary efficacy in dogs but inconsistent association with NK cell numbers and related gene signatures as biomarkers of clinical effects. Changes in NK cells are more likely to be seen in the TME rather than in peripheral blood, especially with intra-tumor immunotherapies (28). These studies also expose the real and ongoing difficulties in identifying canine NK cells, with virtually every trial using different markers.

The future of canine NK immunotherapy is likely a combinatorial approach that enhances multiple anti-tumor methods. Several groups have spearheaded combination radioimmunotherapy and chemoimmunotherapy trials in dogs with spontaneous cancer with varying involvement of NK cells or related cytokines and genes (29, 36).

Four dogs with advanced stage melanoma were treated with trimodal immuno-radiotherapy which included sub-ablative external beam radiation therapy (EBRT), targeted radionuclide therapy (TRT), and intratumor immunocytokine (IT-IC) (37). Numbers of NK cells in circulating blood identified by flow cytometry using CD3−CD5^dim^ did not change significantly with treatment (37). However, RNA-seq analysis of tumor tissue from dogs before and following treatment indicated significant upregulation of KLRA1, KLRB1, NCR3 IL18R1, and TNFα at selected timepoints (37). The small sample size precludes conclusions regarding survival outcomes but may aid in contextualizing the contribution of NK cells in response to therapy.

The importance of placing immune changes in the framework of progression or survival is well-illustrated in an unrelated trial which enrolled 18 dogs with B cell lymphoma that were treated with doxorubicin chemotherapy, anti-CD20 monoclonal antibody, and a small molecule inhibitor (38). Lymph node aspirates analyzed by RNA-seq demonstrated genes associated with NK function as the most significantly upregulated gene set in dogs with poor survival, but samples were obtained from only one time point limiting conclusions regarding immune changes in response to therapy (38). Serial sampling of patient lymph nodes and tracking of changes in response to treatment would help clarify the conclusions of the study and identify predictive in addition to prognostic biomarkers.

Prognostic biomarkers of response were similarly investigated in a trial treating dogs with oral malignant melanoma using checkpoint blockade (39). The Ohashi Laboratory pioneered PD-L1 antibody therapy in dogs and have completed two clinical trials to date (40–42). The mechanism of anti-PD-L1 therapy is based on the understanding that PD-L1 on tumor cells binds to PD-1 on T cells, providing an inhibitory signal that interferes with anti-tumor T cell functions. In the context of NK cells, binding of anti-PD-L1 antibody to PD-L1 expressed on NK cells can increase activation and effector function (43). Thus, the treatment has the potential to both remove T cell inhibition and enhance NK cell function. While the initial canine anti-PD-L1 trial publications did not include immune correlates of response, a follow-up investigation of serum biomarkers by the same group found that overall survival following treatment was positively correlated with low PGE2, higher IL-2, and higher IL-12 in pre-treatment sera, helping to identify COX-2 as a potential target for future trials (39). NK cells were not the primary focus of the trial, but the authors noted that PGE2 is capable of suppressing the function of NK cells and IL-12 is well-established as necessary for the release of IFNγ by NK cells, the most prominent producers of the inflammatory cytokine (39). These studies illustrate the potential for clinical trials to inform future studies, identifying dogs exhibiting high baseline PGE2 serum levels as candidates for the addition of a COX-2 inhibitor. Given the successful development of anti-canine PD-1/L1 antibodies, determining whether these and other immune checkpoints are found on canine NK cells would have a profound impact on prospective targets and combinatorial approaches. Taken together, these data provide preliminary support for future investigations into combination NK immunotherapies to holistically impart anti-tumor effects.

## Discussion

NK immunotherapy in dogs is progressing at an escalating rate with larger sample sizes and collaboration between university hospitals and specialty centers. At the time of this publication, the American Veterinary Medical Association (AVMA) Animal Health Studies Database lists five trials currently recruiting, and 35 trials with completed recruitment based on a search for “immunotherapy” in dogs with cancer. Studies from the University of Minnesota have demonstrated increased Antibody-Dependent Cellular Cytotoxicity (ADCC) efficacy in engineered human NK cells expressing recombinant CD64, opening the doors for the development of engineered canine NK cells that have similar effector capabilities (44). Investigators at The Ohio State University have simultaneously made progress in attempting to improve adoptive NK cell products for canine immunotherapy by imprinting NK cells with TGF-β during expansion to override potential inhibition in the TME (45). This group is using this method of TGFβ-imprinted NK cell therapy combined with carboplatin chemotherapy to treat dogs with OSA in an ongoing phase I clinical trial. Our own group has sought to improve the canine NK product using the expansion of unmanipulated PBMCs from healthy donors for allogeneic adoptive NK cell transfer. These works provide the infrastructure from which canine NK cells can be manipulated to enhance persistence and efficacy in future immunotherapy trials and this multi-institutional, rapid innovation in canine NK immunotherapy is indicative of the growing interest and recognized potential in the field.

By reviewing recent trials with available NK cell correlates, we begin to elucidate an intricate framework of NK responses to treatment. Overall, there is evidence of both NK activation and inhibition in canine immunotherapy with moderate and irregular impacts on NK cell proportions which vary based on intratumor versus peripheral sampling. Timing of sampling is also highly relevant, given that NK correlation to improved response can be negative or positive based on the treatment stage, a concept that can be expected in the context of limited NK cell half-life. Resolution of the role of NK cells in canine immunotherapy requires additional trials with intra-tumor and peripheral immune serial sampling and adequate enrollment for response assessments. The current literature clearly points to the potential promise of NK cell targeting, especially in combination therapies, to benefit both dogs and people for whom novel immunotherapies are needed.

## Author contributions

AR: Writing – original draft, Writing – review & editing. AG: Writing – original draft, Writing – review & editing. CT: Writing – review & editing. RR: Writing – review & editing. WM: Writing – review & editing. MK: Writing – review & editing. RC: Writing – original draft, Writing – review & editing.
